# Progression patterns in monoclonal gammopathy of undetermined significance and multiple myeloma outcome: a cohort study in 42 patients

**DOI:** 10.1186/s40164-022-00259-0

**Published:** 2022-02-23

**Authors:** Widad Tahiru, Antonio Izarra Santamaria, Johan Hultdin, Wendy Yi-Ying Wu, Florentin Späth

**Affiliations:** 1 Department of Radiation Sciences, Oncology, Umeå University, 90187 Umeå, Sweden; 2 Department of Radiation Sciences, Oncology and Cancer Center, Hematology, Umeå University, 90187 Umeå, Sweden; 3 Department of Medical Biosciences, Clinical Chemistry, Umeå University, Umeå, Sweden

**Keywords:** Multiple myeloma, Myeloma outcome, Aggressive myeloma, MGUS, Low-risk MGUS, MGUS follow-up, MGUS progression, Prospective blood samples, NSHDS

## Abstract

Follow-up of low-risk monoclonal gammopathy of undetermined significance (MGUS) is debated as multiple myeloma (MM) progression risk is low. Worse MM outcome was reported for patients followed for low-risk MGUS, possibly due to less optimal follow-up. However, it is unknown whether progressing low-risk MGUS is associated with aggressive tumor behavior. Understanding these patterns is crucial for MGUS management. Here, we investigated whether progression from low-risk MGUS is associated with worse MM outcome in patients who had no MGUS follow-up before myeloma diagnosis. We retrospectively determined the MGUS status in repeated pre-diagnostic blood samples prospectively collected from 42 myeloma patients in median 11.6 years (first sample) and 3.3 years (repeated sample) before myeloma diagnosis. At first pre-diagnostic blood draw, 12 had low-risk (defined by an immunoglobulin [Ig] G monoclonal [M] spike < 15 g/L and a normal free light-chain ratio) and 30 had MGUS of other risk. MM bone disease was more common in patients with low-risk MGUS at first blood draw (67% vs. 30%, P = 0.041). Median survival since myeloma diagnosis was worse in low-risk than other MGUS at first blood draw (2.3 vs. 7.5 years, P = 0.004). Modest progression was observed between first and repeated blood draw for the majority of low-risk MGUS as 67% remained as low- or low-intermediate-risk MGUS at repeated blood draw. Our study, albeit limited by its small size, indicates that progression from low-risk MGUS is associated with worse MM outcome regardless of MGUS follow-up. Although further investigation is needed, progressing low-risk MGUS could belong to a group of aggressive tumors with progression that is difficult to predict.

**Supplementary Information:**

The online version contains supplementary material available at 10.1186/s40164-022-00259-0.

## To the editor

Multiple myeloma (MM) is preceded by monoclonal gammopathy of undetermined significance (MGUS) [1, 2]. Guidelines recommend following MGUS according to MM progression risk [3]. Follow-up of low-risk MGUS is debated as progression risk is low (5% at 20 years) [4]. Studies evaluating MGUS follow-up indicate worse MM outcome in patients followed for low-risk MGUS, possibly due to less optimal follow-up [5, 6]. However, it is unknown whether progressing low-risk MGUS is associated with aggressive tumor behavior. Understanding these patterns is important for MGUS management [7]. Therefore, the association between progressing low-risk MGUS and MM outcome needs further study. We investigated whether progression from low-risk MGUS is associated with worse MM outcome in patients who had no MGUS follow-up before myeloma diagnosis. The MGUS status was determined retrospectively in repeated pre-diagnostic blood samples of 42 myeloma patients.

The Umeå University review board approved this study using samples from the Northern Sweden Health and Disease Study, a large prospective cohort. Linkage to the Swedish Cancer Registry facilitated identification of myeloma patients with a first and repeated pre-diagnostic blood sample before myeloma diagnosis. We could study natural progression patterns in relation to MM outcome because 42 had detectable MGUS (protein and immunofixation electrophoresis and free light-chain assays) in both pre-diagnostic samples without MGUS follow-up before myeloma diagnosis. Kaplan–Meier plots and multivariable Cox regression were used to study overall survival (Additional file 1).

The first pre-diagnostic blood sample was donated in November 1986 and the last follow-up since myeloma diagnosis was in February 2021 providing a 19-year study duration in median. Median times since first and repeated pre-diagnostic blood draw to myeloma diagnosis were 11.6 and 3.3 years (Table 1). At first pre-diagnostic blood draw, 12 had low-risk (defined by immunoglobulin [Ig] G monoclonal [M] spike < 15 g/L and normal free light-chain ratio) and 30 had MGUS of any other risk category (i.e., low-intermediate-risk, high-intermediate-risk, high-risk, or light-chain MGUS) (Table 1). Male sex was more common in patients with low-risk MGUS at first blood draw. Other characteristics, including age, diagnosis year, comorbidities, myeloma therapy, and access to novel drugs, were similar in both groups (Table 1, Additional File 2: Table S1–2).

**Table 1:** Characteristics of the study population by MGUS risk at first pre-diagnostic blood draw

Characteristic	Low-risk MGUS^a^ N (%)	Other MGUS^b^ N (%)	P value^c^
Total	12 (100)	30 (100)	–
Median years to myeloma diagnosis (range)			
First pre-diagnostic blood draw	13.5 (6.8–18.7)	11.1 (1.5–19.3)	0.15
Second pre-diagnostic blood draw	4.2 (0.2–11.6)	3.0 (0.5–14.3)	0.20
Median age at myeloma diagnosis in years (range)	61 (48–84)	62 (51–79)	0.52
Sex			
Female	7 (58)	26 (87)	0.09
Male	5 (42)	4 (13)	
Isotype			
IgG	12 (100)	15 (50)	
Non-IgG (IgA and IgD)	–	7 (23)	
Light-chain	–	8 (27)	
International staging system (ISS) stage			
ISS 1	5 (42)	20 (67)	0.17
ISS-2 or ISS-3	7 (58)	10 (33)	
Disease status at myeloma diagnosis			
Multiple myeloma (MM)	10 (83)	17 (57)	0.16
Smoldering multiple myeloma (SMM)	2 (17)	13 (43)	
Bone disease at myeloma diagnosis^d^			
Presence of MM bone disease	8 (67)	9 (30)	0.04
Absence of MM bone disease	4 (33)	21 (70)	
Imaging at myeloma diagnosis			
Only conventional skeletal survey	7 (58)	20 (67)	0.73
Additional imaging modalities^e^	5 (42)	10 (33)	
Performance status			
ECOG 0–1	11 (92)	26 (87)	1.00
ECOG 2–3	1 (8)	4 (13)	
Diagnosis calendar period			
1997–2003	4 (33)	9 (30)	
2004–2007	3 (25)	13 (43)	0.49
2008–2012	5 (42)	8 (27)	
Median % clonal plasma cells (range)	30 (10–80)	21 (8–80)^f^	0.20
Hemoglobin^g^			
Normal	3 (25)	16 (53)	0.17
Below normal	9 (75)	14 (47)	
Creatinine^h^			
Normal	7 (58)	23 (77)	0.27
Above normal	5 (42)	7 (23)	
Corrected calcium			
Normal (2.15–2.50 mmol/L)	11 (92)	22 (73)	0.25
Above normal (> 2.50 mmol/L)	1 (8)	8 (27)	
Beta-2-microglobulin			
Normal (0.7–1.9 mg/L)	2 (17)	4 (13)	1.00
Above normal (> 1.9 mg/L)	10 (83)	26 (87)	
Lactate dehydrogenase^i^			
Normal	9 (75)	21 (70)	1.00
Above normal	3 (25)	9 (30)	

^a^Defined by IgG M spike < 15 g/L and normal free light-chain ratio at first pre-diagnostic blood draw

^b^Low-intermediate-risk (N = 11), high-intermediate-risk (N = 10), high-risk (N = 1), or light-chain (N = 8) MGUS at first blood draw

^c^Fisher’s exact test was used to compare categorical variables and the Mann–Whitney U test was used for continuous variables

^d^Defined as osteolytic lesions and/or vertebral compression fractures due to the underlying multiple myeloma

^e^Additionally performed imaging modalities included computed tomography and/or magnetic resonance imaging

^f^One patient with 8% clonal bone marrow plasma cells fulfilled myeloma criteria based on urine electrophoresis

^g^Normal range in women 117–153 g/L; normal range in men 134–170 g/L

^h^Normal range in women 45–90 µmol/L; normal range in men 60–105 µmol/L

^i^Normal value in individuals 18–70 years < 3.4 µkat/L; normal value in individuals > 70 years < 4.2 µkat/L

**Fig. 1:**
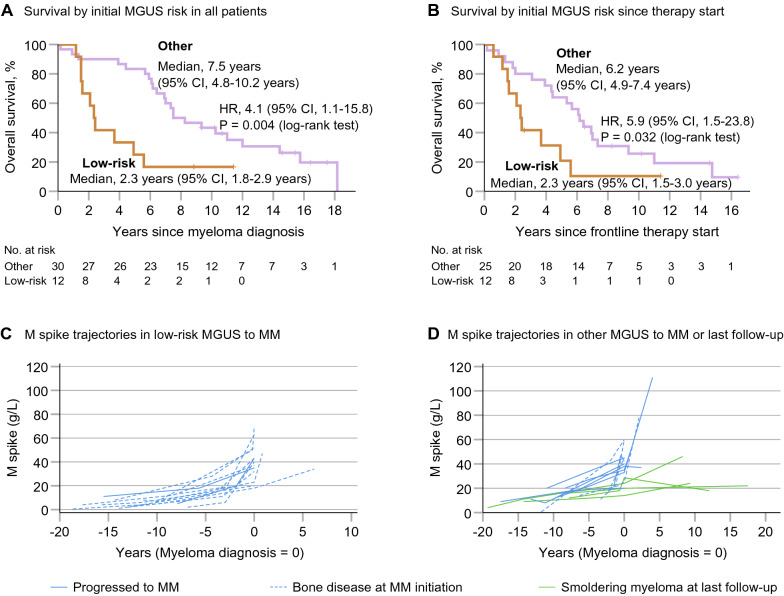
MGUS (low risk vs. other MGUS at first pre-diagnostic blood draw) progressing to myeloma. **A** Overall survival since myeloma diagnosis. Hazard ratios (HRs) and 95% confidence intervals (CIs) for death adjusted for sex, age at diagnosis (continuous), time of diagnosis (continuous), Eastern Cooperative Oncology Group (ECOG) performance status (ECOG 0 or 1 vs. ECOG 2 or 3), International Staging System (ISS) stage (ISS-1 vs. ISS-2 or ISS-3), the proportion of clonal bone marrow plasma cells (continuous), lactate dehydrogenase levels (normal vs. elevated), disease status at myeloma diagnosis (smoldering multiple myeloma [SMM] vs. multiple myeloma [MM]), and immunoglobulin (Ig) isotype (IgG vs. non-IgG vs. light-chain). **B** Overall survival since frontline therapy start excluding five patients who did not progress to MM. HRs and 95% CIs for death adjusted for sex, age at diagnosis (continuous), time of diagnosis (continuous), performance status (ECOG 0 or 1 vs. ECOG 2 or 3), ISS stage (ISS-1 vs. ISS-2 or ISS-3), the proportion of clonal bone marrow plasma cells (continuous), lactate dehydrogenase levels (normal vs. elevated), isotype (IgG vs. non-IgG vs. light-chain), and treatment details (autologous stem cell transplant [ASCT] vs. no ASCT; proteasome inhibitor [PI] and immunomodulating drug [IMiD] vs. PI or IMiD vs. no modern drug; modern drug in frontline treatment vs. not). **C**, **D** M spike trajectories in patients who had low-risk MGUS (N = 12) and other MGUS of IgG isotype (N = 15) at first pre-diagnostic blood draw (for better comparison restricted to IgG isotype). M spike concentrations are plotted for each individual at the time point of first and at repeated pre-diagnostic blood draw, at myeloma diagnosis (which is indicated by the time point 0), and at MM initiation (i.e. frontline therapy start) or the time of last clinical follow-up in four individuals who did not progress to MM

At myeloma diagnosis, 83% vs. 57% had symptomatic MM in patients who had low-risk vs. other MGUS, respectively, at first pre-diagnostic blood draw (P = 0.158) (Table 1). Excluding light-chain myeloma (N = 8), formal statistical significance was reached (83% vs. 41% [9 of 22], P = 0.030). At myeloma diagnosis, bone disease (osteolytic lesions and/or vertebral compression fractures due to MM) was more common in low-risk vs. other MGUS at first blood draw (P = 0.041; Table 1). This was pronounced excluding light-chain myeloma (P = 0.008). Imaging along conventional skeletal surveys was similarly used in both groups (Table 1). In low-risk vs. other MGUS, median survival since myeloma diagnosis was 2.3 years vs. 7.5 years (Fig. 1A). Results were similar for survival since therapy start and in multivariable analyses (Fig. 1A, B). Sex was not associated with bone disease and survival. The results were confirmed in several sensitivity analyses (Additional file 1).

We compared MM progression trajectories in patients who had low-risk vs. other MGUS (restricted to IgG isotype for better comparison) at first pre-diagnostic blood draw. At repeated pre-diagnostic blood draw, progression to smoldering multiple myeloma (M spike ≥ 30 g/L) was observed in 8% (1 of 12) in low-risk vs. 20% (3 of 15) in other MGUS (P = 0.605). More patients with low-risk MGUS at first pre-diagnostic blood draw had lower MGUS risk (low- or low-intermediate-risk) at repeated blood draw compared to other MGUS (67% [8 of 12] vs. 27% [4 of 15], P = 0.057). This was pronounced excluding four patients who did not progress to symptomatic MM (all had other MGUS at first blood draw; 67% vs. 9% [1 of 11], P = 0.009). These observations could indicate a more rapid progression process in low-risk MGUS closer to MM initiation. Investigating this, we plotted M spikes in both groups. M spike trajectories were visually largely similar in both groups with some patients experiencing rapid clonal evolution as indicated by fast increasing paraprotein levels (Fig. 1C, D). The annual median M spike increase since repeated pre-diagnostic blood draw was 6.0 g/L in low-risk and 2.2 g/L in other MGUS (P = 0.13) (Fig. 1C, D, Additional File 2: Table S3).

Our study, which included data collected over 19 years in median with 81% treated using novel drugs and overall survival comparable to other studies [8], shows that progression from low-risk MGUS is associated with worse MM outcome. These results agree with previous data [5, 6]; however, we found progressing low-risk MGUS associated with worse MM outcome in patients who had no MGUS follow-up before myeloma diagnosis. Thus, we speculate that progressing low-risk MGUS could belong to a group of more aggressive tumors. Biological mechanisms for this putative association are unclear. Interestingly, substantial genomic differences in patients with stable and progressive myeloma precursor condition have been observed [9] with distinct genomic patterns of progression (“static progression” vs. “spontaneous evolution”) in patients who progressed from SMM to MM [10]. Consistent with recent data [11], progression would have been difficult to predict in many of the low-risk patients as 67% remained low- or low-intermediate-risk MGUS at repeated blood draw (donated in median 7.5 years after the first sample). These observations illustrate limitations of the current MGUS stratification: (i) reduced sensitivity in the accurate identification of low-risk MGUS and (ii) current biomarkers do not predict the biological behavior of the later diagnosed tumor. Accurate early prediction of disease progression and/or aggressive tumor behavior could facilitate the identification of patients potentially benefiting from early therapeutic intervention such as currently under evaluation in high-risk MGUS and low-risk SMM [12].

We speculate that while low-risk MGUS patients are less likely to develop MM, there is a subset of these patients who will progress and, importantly, in case of progression belong to a group of more aggressive tumors. As this study has a small sample size with cytogenetic information only available in 17% of the patients and the most recent IMWG criteria [13] were not applicable, the results require further investigation. Until ongoing studies provide answers [14], our data stress the need for improved MGUS stratification based on specific molecular features rather than biomarkers largely reflective of tumor burden [15]. Investigation of microenvironmental differences in prospective blood samples among stable and progressing MGUS could help (i) increase the understanding of underlying extrinsic factors in MM progression and (ii) identify useful biomarkers.

## Supplementary Information

**Additional file 1:** Patients and methods.

**Additional file 2:** **Table S1.** Comorbidities in patients who had low-risk vs. other MGUS at first pre-diagnostic blood draw. **Table S2.** Myeloma treatment in patients who had low-risk vs. other MGUS at first pre-diagnostic blood draw. **Table S3.** M spike concentrations in low-risk vs. other MGUS of IgG isotype (for better comparison) at first blood draw.

## Data Availability

All data are available in the manuscript or the additional file materials.

## Abbreviations

ASCT: Autologous stem cell transplant
CI: Confidence interval
ECOG: Eastern Cooperative Oncology Group
HR: Hazard ratio
Ig: Immunoglobulin
IMiD: Immunomodulatory drugs
IMWG: International Myeloma Working Group
ISS: International Staging System
MGUS: Monoclonal gammopathy of undetermined significance
MM: Multiple myeloma
M (spike): Monoclonal (spike)
PI: Proteasome inhibitor
SMM: Smoldering multiple myeloma
