# Supplementary material for: Progression patterns in monoclonal gammopathy of undetermined significance and multiple myeloma outcome: a cohort study in 42 patients
Source: Exp Hematol Oncol. 2022 Feb 23;11:8. doi: 10.1186/s40164-022-00259-0 (PMC8867673; doi:10.1186/s40164-022-00259-0)
Supplement: Supplementary file 1 — Additional file 1: Patients and methods. [file 40164_2022_259_MOESM1_ESM.docx]

**Patients and Methods**

Study cohort

The Northern Sweden Health and Disease Study (NSHDS) is a prospective longitudinal cohort with more than 100,000 individuals [1]. NSHDS consists of three sub-cohorts: the Västerbotten Intervention Program (VIP), the Mammary Screening Project (MA), and the Northern Sweden Monica Project. For this study, participants of VIP and MA were available for inclusion. VIP is a population-based cohort of individuals 40, 50, and 60 years of age who have donated blood samples within health surveys since 1985. These health surveys were initiated to reduce morbidity from cardiovascular disease and diabetes by inviting all individuals to participate in systematic risk factor screening. MA is a cohort of females attending mammary screening with repeated blood samples available between 1995 and 2006. Linkage of both NSHDS cohorts (VIP and MA) to the Swedish Cancer Registry facilitated the identification of myeloma patients who had donated two pre-diagnostic blood samples before myeloma diagnosis (N = 61). These repeated pre-diagnostic blood samples (N = 122) were retrieved and evaluated for presence of monoclonal gammopathy of undetermined significance (MGUS) by using protein and immunofixation electrophoresis and free light-chain assays. Of 61 individuals, 45 had detectable MGUS in both pre-diagnostic samples. Three subjects were excluded because they had MGUS follow-up before myeloma diagnosis. Thus, 42 participants were eligible for inclusion in the present study to investigate natural progression patterns. All participants in this study (N = 42) were part in an earlier nested case-control study investigating cytokine and growth factor trajectories in future myeloma patients [2]. The Umeå University review board approved this study (2017/242-31).

MGUS assessment

Protein and immunofixation electrophoresis were used to detect M proteins (Sebia, France) and a Cobas 8000 analyzer (Roche, Germany) was used to measure immunoglobulin and free light-chains with the Freelite assay (The Binding Site, UK). For all included participants (N = 42), MGUS risk was determined blinded to the identity of the samples according to criteria of the Mayo Clinic [3].

Clinical data collection

The most recent IMWG criteria were not applicable because all patients (N = 42) were diagnosed before 2014. Thus, all patients were classified by the treating physician into multiple myeloma (MM) or smoldering myeloma based on earlier IMWG criteria [4]. All clinical data were collected independently by two of the investigators. In case of inconsistencies, original source files were reassessed. Potential differences between groups (low-risk vs. other MGUS) regarding comorbidities and provided treatment were rigorously evaluated. Six patients had missing values for blood levels of lactate dehydrogenase and one patient had a missing Beta-2-microglobulin level at myeloma diagnosis. Cytogenetic information was only available in 17% of the patients and was therefore not evaluated.

Statistical analyses

Overall survival was defined as the time from myeloma diagnosis to death or the date of last follow-up. Overall survival was determined using the Kaplan-Meier method and the log-rank test. Hazard ratios (HRs) and 95% confidence intervals (CIs) were calculated using multivariable Cox regression adjusted for known prognostic factors. The proportional-hazard assumption was assessed as described previously [5], without finding an indication of violation. To avoid omission of patients with missing values for lactate dehydrogenase (N = 6) and Beta-2-mikroglobulin (N = 1) in multivariable analyses, we used multiple imputation [6]. All statistical tests were two-sided and all analyses were performed using IBM SPSS Version 26.0.

**Sensitivity analyses**

To evaluate our results further, we performed different sensitivity analyses. Male sex was more common in patients who had low-risk (42%) compared to other MGUS (13%) at first pre-diagnostic blood draw (P = 0.09). As indicated in the manuscript text, we did not find a significant association between sex and bone disease (P = 0.12) or sex and overall survival (P = 0.49). Nevertheless, the unequal distribution of sex between both groups, could have influenced the results. To evaluate whether worse outcome in low-risk MGUS was driven by the overrepresentation of men, we repeated all analyses excluding male participants (N = 9). Results of these analyses did not support the presence of such an effect. Bone disease (attributable to MM) at myeloma diagnosis was more common in patients who had low-risk MGUS at first pre-diagnostic blood draw (P = 0.03). Median survival since myeloma diagnosis was 2.3 vs. 7.5 years in low-risk vs. other MGUS at first blood draw (P < 0.001).

Accounting for the potential influence of competing causes of death on the result, we repeated survival analysis using MM-specific survival as the endpoint. Results from this analysis confirmed the results from overall survival analysis with a significantly shorter MM-specific survival in patients with low-risk compared to other MGUS at first blood draw (2.4 years vs. 15.8 years, P = 0.002). To evaluate the influence of multiple imputation in multivariable models, we excluded patients with missing values for lactate dehydrogenase (N = 6) and Beta-2-microglobulin (N = 1). Results from these analyses also remained similar compared to results from the main model including all study participants (N = 42).

The distribution of NSHDS sub-cohorts included in this study (VIP and MA) was similar in patients with low-risk and other MGUS at first pre-diagnostic blood draw (P = 0.24). However, as intervention due to general health surveys (VIP) and mammography screening (MA) could have introduced bias, we performed further sensitivity analyses. First, we excluded all participants who underwent a general health survey (VIP) at both pre-diagnostic blood draws. Excluding these participants (N = 15), results remained similar. Bone disease at myeloma diagnosis was more common in patients with low-risk MGUS at first pre-diagnostic blood draw (P = 0.09) and median survival since myeloma diagnosis was 2.1 vs. 7.5 years in low-risk vs. other MGUS (P < 0.001). In addition, we performed analyses excluding individuals who had donated blood samples within two years of myeloma diagnosis (these had either an appointment for a general health survey or mammography screening in the two years prior to myeloma diagnosis). Excluding these participants (N = 13), results remained the same. Bone disease at myeloma diagnosis was more common in patients with low-risk MGUS at first blood draw (P = 0.05). Median survival since myeloma diagnosis was 2.3 vs. 10.3 years in low-risk vs. other MGUS (P = 0.04).

**References**

1. Hallmans G, Ågren Å, Johansson G, Johansson A, Stegmayr B, Jansson J-H, et al. Cardiovascular disease and diabetes in the Northern Sweden Health and Disease Study Cohort- evaluation of risk factors and their interactions. Scandinavian Journal of Public Health 2003, 31(61 suppl):18-24.

2. Späth F, Wibom C, Krop EJM, Santamaria AI, Johansson A-S, Bergdahl IA, et al. Immune marker changes and risk of multiple myeloma: a nested case-control study using repeated pre-diagnostic blood samples. Haematologica 2019, 104(12):2456-2464.

3. Rajkumar SV, Kyle RA, Buadi FK. Advances in the Diagnosis, Classification, Risk Stratification, and Management of Monoclonal Gammopathy of Undetermined Significance: Implications for Recategorizing Disease Entities in the Presence of Evolving Scientific Evidence. Mayo Clinic Proceedings 2010, 85(10):945-948.

4. The International Myeloma Working G: Criteria for the classification of monoclonal gammopathies, multiple myeloma and related disorders: a report of the International Myeloma Working Group. British Journal of Haematology 2003, 121(5):749-757.

5. Delgado J, Pereira A, Villamor N, López-Guillermo A, Rozman C. Survival analysis in hematologic malignancies: recommendations for clinicians. Haematologica 2014, 99(9):1410-1420.

6. Lubin JH, Colt JS, Camann D, Davis S, Cerhan JR, Severson RK, et al. Epidemiologic Evaluation of Measurement Data in the Presence of Detection Limits. Environmental Health Perspectives 2004, 112(17):1691-1696.
